# Association of the Respiratory Severity Score with Bronchopulmonary Dysplasia-Associated Pulmonary Hypertension in Infants Born Extremely Preterm

**DOI:** 10.21203/rs.3.rs-2852392/v1

**Published:** 2023-04-27

**Authors:** Matthew Kielt, Lindsey Beer, Brian Rivera, Waceys Jama, Jonathan Slaughter, Carl Backes, Sara Conroy

**Affiliations:** Nationwide Children’s Hospital, The Ohio State University College of Medicine; Nationwide Children’s Hospital; Abigail Wexner Research Institute at Nationwide Children’s Hospital; The Ohio State University; Nationwide Children’s Hospital; The Ohio State University

**Keywords:** bronchopulmonary dysplasia, bronchopulmonary dysplasia-associated pulmonary hypertension, echocardiography

## Abstract

**Objective:**

To test the hypothesis that elevations in the respiratory severity score (RSS) are associated with increased risk of bronchopulmonary dysplasia-associated pulmonary hypertension (BPD-PH).

**Study Design:**

Retrospective cohort study of infants born extremely preterm admitted to a BPD center between 2010–2018. Echocardiograms obtained ≥36 weeks’ post-menstrual age (PMA) were independently adjudicated by two blinded cardiologists to determine the presence/absence of BPD-PH. Multivariable logistic regression estimated the association between RSS with BPD-PH.

**Result:**

BPD-PH was observed in 68/223 (36%) of subjects. The median RSS at time of echocardiography was 3.04 (Range 0–18.3). A one-point increase in RSS was associated with BPD-PH, aOR 1.3 (95% CI 1.2–1.4), after adjustment for gestational age and PMA at time of echocardiography.

**Conclusion:**

Elevations in the RSS were associated with a greater risk of BPD-PH. Prospective studies are needed to determine the validity and performance of RSS as a clinical susceptibility/risk biomarker for BPD-PH.

## Introduction

Bronchopulmonary dysplasia (**BPD**) is the most common morbidity experienced by infants born extremely preterm.^1^ Approximately 20–40% of infants with BPD develop pulmonary vascular disease, which manifests clinically as BPD-associated pulmonary hypertension (**BPD-PH**).^2–5^ The risk of BPD-PH is inversely proportional to gestational age at birth and a diagnosis of BPD-PH is associated with short- and long-term morbidity with mortality estimates ranging from 12–38%.^2, 4, 6–8^ Given the risk of morbidity and mortality associated with BPD-PH,^9^ consensus guidelines recommend obtaining a screening echocardiogram at 36 weeks’ post-menstrual age (**PMA**), which corresponds to the time of formal BPD diagnosis.^10, 11^ Nonetheless, echocardiogram utilization for the diagnosis of BPD-PH varies from 26–86% between United States Children’s Hospitals neonatal intensive care units (**NICUs**)^4^ and the optimal timing of repeat BPD-PH echocardiography after 36 weeks’ PMA remains unknown.

Underutilization of echocardiograms to evaluate for BPD-PH may result in delayed diagnosis, delayed initiation of therapy, and increased morbidity for patients. However, the clinical utility of echocardiographic assessment of BPD-PH may be limited^12^ and overutilization of this diagnostic tool may result in unnecessary exposure to invasive diagnostics, including cardiac catheterization,^12^ and increased costs related to clinician-driven tests.^13^ Consequently, there is a real need for improved precision regarding the utilization of echocardiography for the evaluation of BPD-PH. Identifying pragmatic BPD-PH susceptibility/risk biomarkers that can be measured non-invasively and at no cost to the patient may plausibly improve BPD-PH risk stratification and reduce variability in echocardiogram utilization in this population.

The respiratory severity score (**RSS**) is a non-invasive biomarker that is equal to the product of mean airway pressure (**MAP**) and the fraction of inspired oxygen (**FiO**_**2**_).^14^ An elevated RSS (modified to account for patients on non-invasive modes of respiratory support) has previously been shown to predict adverse in-hospital outcomes for infants with established severe BPD.^15^ Whether an elevated RSS is associated with an increased risk for BPD-PH remains unknown. Therefore, in this study, our objective was to test the hypothesis that elevations in the RSS, measured concurrent with echocardiography, are associated with increased risk of BPD-PH in infants born extremely preterm.

## Materials/Subjects and Methods

We performed this retrospective cohort study in accordance with Strengthening the Reporting of Observational Studies in Epidemiology guidelines.^16^ Included patients were born < 28 weeks’ gestation who were admitted to the 24-bed BPD unit at Nationwide Children’s Hospital (**NCH**) between January 1, 2010 and December 31, 2018. Included patients had diagnosis of severe BPD, as defined by 2001 National Institutes of Health Consensus criteria,^10^ which were the criteria for enrollment into our BPD program during the study period. Included subjects had at least one echocardiogram performed at or after 36 weeks’ PMA (+/− 1 week). Excluded patients were those with congenital heart disease (excluding atrial septal defect, ventricular septal defect, and patent ductus arteriosus), a genetic syndrome or aneuploidy, a major congenital malformation, and those patients with missing clinical data needed to calculate the RSS. Clinical characteristics of included patients were gathered from the medical record. The Institutional Review Board at NCH approved this study with a waiver of consent.

We calculated the RSS using respiratory support data that were readily available at the time of echocardiography. As described in prior studies, we calculated the RSS on subjects treated with invasive mechanical ventilation, non-invasive positive airway pressure, low-flow nasal cannula, and room air.^17^ For subjects treated with invasive mechanical ventilation, the RSS is equal to the product of MAP and FiO_2_ where MAP was calculated based on prescribed ventilator settings based on the following equation: $\text{MAP}=\text{PEEP}+\left[\left(\text{PIP}-\text{PEEP}\right)\times \left({\text{t}}_{\text{i}}/{\text{t}}_{\text{i}}+{\text{t}}_{\text{e}}\right)\right]$ and **PEEP** is the positive end-expiratory pressure, **PIP** is the peak inspiratory pressure, **t**_**i**_ is the inspiratory time, and **t**_**e**_ is the expiratory time. Briefly, to calculate the RSS for patients on non-invasive positive pressure ventilation, we assumed $\text{MAP}=\text{PEEP}+\left(\left(\text{PIP}-\text{PEEP}\right)\times \left({\text{t}}_{\text{i}}/({\text{t}}_{\text{i}}+{\text{t}}_{\text{e}}\right)\right)$, for patients on continuous positive airway pressure (**CPAP**), we assumed MAP= CPAP, for patients on high-flow nasal cannula ≥ 2 liters per minute (**LPM**) we assumed MAP=flow in LPM, for patients on low-flow nasal cannula < 2 LPM, we assumed MAP=1 and for patients on room air, we assumed MAP = 0.^17^

The primary outcome for this study was echocardiographic evidence of BPD-PH ≥ 36 weeks’ PMA, defined dichotomously (yes/no). At our center, infants born extremely preterm with severe BPD were screened for BPD-PH at approximately 36 weeks’ PMA. For infants with no prior history of BPD-PH, repeat screening echocardiograms were performed every 1–2 months until hospital discharge. For infants with BPD-PH, echocardiograms were typically obtained every 1–2 weeks until resolution of BPD-PH, defined as 2 consecutive echocardiograms without evidence of BPD-PH. Consistent with previous studies,^8, 17^ BPD-PH was determined as a binary variable using the following hierarchy: 1) estimated right ventricular systolic pressure (**RVSP**) greater than 40 mmHg; 2) RVSP/systemic systolic blood pressure greater than 0.5; 3) any cardiac shunt with bidirectional or right-to-left flow; 4) or any degree of ventricular septal wall flattening. RVSP was determined from the tricuspid regurgitation jet velocity using the modified Bernoulli equation and assuming a right atrial pressure of 5 mm Hg.^8, 18^ During the study period, brain natriuretic peptide (**BNP**) and N-terminal pro b-type natriuretic peptide (**NT-pro-BNP**) were not routinely assayed at the time of BPD-PH evaluation. Determination of BPD-PH was made following independent adjudication of clinical echocardiograms by 2 blinded pediatric cardiologists. Disagreements regarding echocardiographic evidence of BPD-PH were settled through discussion, with involvement of a third reviewer, as necessary.

### Statistical Analysis

Continuous data had skewed distributions and are summarized using median and interquartile range (**IQR**). Categorical data are presented as number and percent. We compared clinical characteristics for the cohort, stratified by the study outcome of at least one echocardiogram demonstrating BPD-PH after 36 weeks’ PMA using Mann-Whitney U and chi-square tests, for continuous and categorical data, respectively. Generalized linear mixed models with logit link and infant as a random effect were used to estimate the association of RSS with BPD-PH. Odds ratios and predicted probabilities of BPD-PH are presented for both unadjusted and adjusted models. Adjusted estimates included gestational age and PMA at the time of echocardiography as fixed effects. BPD severity was not included as a covariate in adjusted models because it is a collider rather than a confounder for the association between RSS and BPD-PH^19^ and prior studies have demonstrated the presence of BPD-PH across the spectrum of BPD disease severity.^7^ Statistical analyses were performed using R version 4.2.0 (R Foundation for Statistical Computing, Vienna, Austria). Odds ratios were considered statistically significant if the 95% confidence interval did not cross 1.

## Results

**Figure 1:**
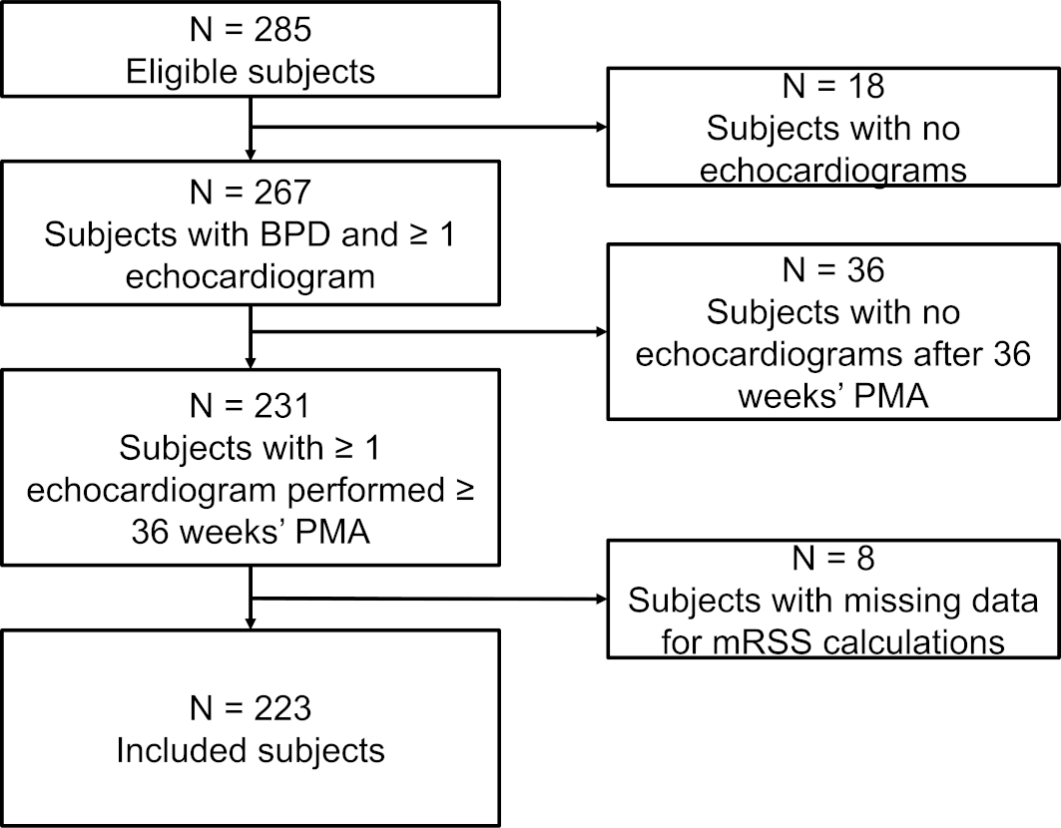
Flow diagram for the cohort.

**Figure 2:**
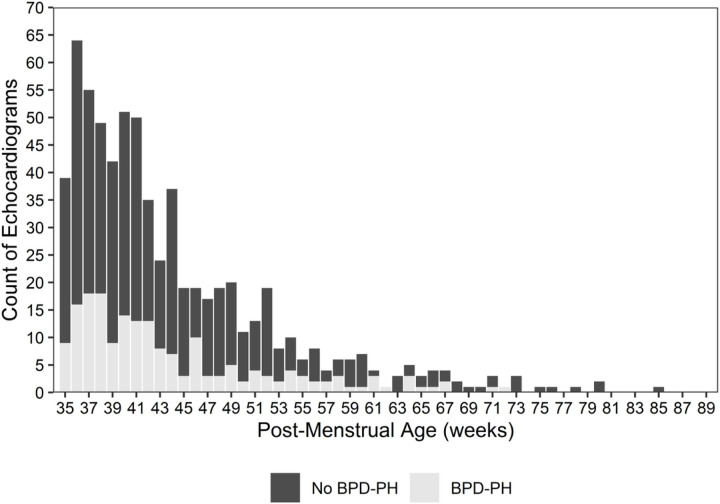
Distribution of timing of echocardiography in the cohort, stratified by BPD-PH status at the time of assessment.

The study cohort included 223 patients (Fig. 1). In these 223 patients, a total of 680 echocardiograms were performed ≥ 36 weeks’ PMA, of which 189/680 (28%) demonstrated evidence of BPD-PH (Fig. 2). Of the 223 included patients, 68 (36%) were diagnosed with BPD-PH.

The clinical characteristics of the cohort are presented in Table 1. In general, members of the cohort were born at extremely low gestational ages, born at extremely low birth weights, and were male. Mortality in this cohort was 4%. As compared to subjects without BPD-PH, subjects with BPD-PH were more likely to be born to mothers with a hypertensive disorder of pregnancy, be born at lower birth weights, SGA, be female, have grade III or IV IVH, have echocardiographic evidence of pulmonary hypertension prior to 36 weeks’ PMA, have longer lengths of stay, and die (Table 1).

**Figure 3:**
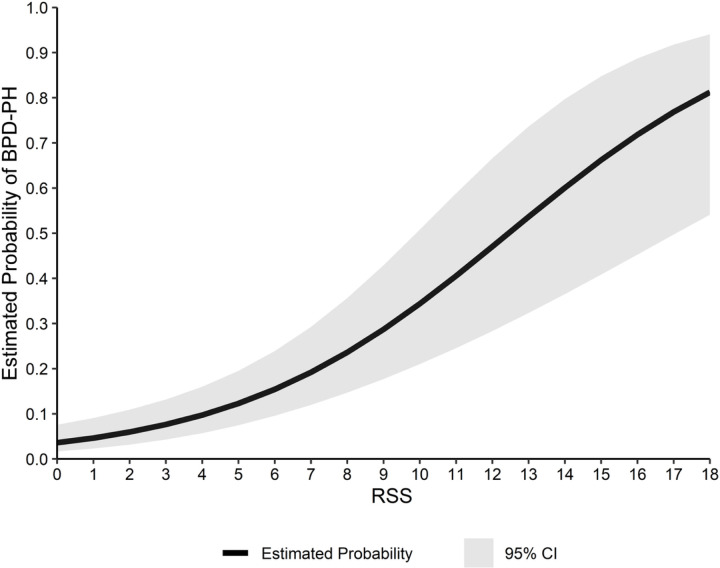
Estimated probabilities of BPD-PH with continuous increases in the RSS. The estimated probabilities were derived from a logistic regression model that accounted for repeat echocardiograms with infant treated as a random effect. The presented model was controlled for gestational age at birth.

We observed an increased odds of BPD-PH for a one-point increase in the RSS (aOR 1.3 [95% CI 1.2, 1.4]) after adjusting for gestational age and PMA at the time of echocardiography, which corresponded to an increased probability of BPD-PH (0.027, 95% CI 0.17, 0.38, Table 2, Model 3). The estimated probability of BPD-PH across observed RSS measurements is shown in Fig. 3. The precision of the estimated probability decreased as the RSS increased due to fewer cohort subjects with elevated levels of the RSS (Fig. 3).

## Discussion

In this study, we observed that an increased RSS, measured concurrent with echocardiography, was associated with an increased risk of BPD-PH in a cohort of infants born extremely preterm. This knowledge is important because infants with BPD-PH are at a 5-fold increased risk of death compared to infants without BPD-PH^9^ and it is critical that infants with BPD are screened for BPD-PH in order to promptly diagnosis and treat the disease.^4, 8, 18, 20^ Echocardiograms are the most frequently used screening assessment for BPD-PH yet echocardiograms are expensive,^13^ may lack specificity for BPD-PH,^12^ and are variably utilized across centers.^4^ Cardiac catheterization is the gold standard for BPD-PH diagnosis^21^ yet this procedure carries procedural risks for infants born extremely preterm with major co-morbidities such as BPD.^22^ Additionally, there are no reliable biomarkers of BPD-PH.^23^ As such, the optimal timing of interval screening for BPD-PH after 36 weeks’ PMA remains unknown. Identifying clinical risk factors that can be measured concurrently with echocardiography and identify infants born extremely preterm who are at the highest risk of BPD-PH may increase precision utilization of echocardiography for the evaluation of BPD-PH and facilitate timely initiation of pulmonary vasodilator therapy.

To our knowledge, this is the first study to identify a pragmatic clinical risk score, measured non-invasively and at no cost to the patient, that is associated with concurrent BPD-PH in infants born extremely preterm. Prior studies investigating BPD-PH clinical risk factors have identified factors that are determined at time intervals that are remote to the timing of formal BPD-PH diagnosis. For example, epidemiologic studies have observed an association of BPD-PH with placental abnormalities causing maternal vascular underperfusion,^24^ extremely preterm birth,^4, 8^ very low birth weight,^4, 8^ intra-uterine growth restriction,^25, 26^ and necrotizing enterocolitis.^8, 27, 28^ To identify a clinical BPD-PH risk factor that could be measured in close proximity to BPD-PH screening, Gentle and colleagues recently compared the frequency and duration of intermittent hypoxemia events in the week prior to BPD-PH assessment for 80 infants born extremely preterm with and without BPD-PH. In this well-designed, prospective case-control study, the authors observed that the duration of intermittent hypoxemic events with oxygen saturations < 70% predicted BPD-PH with an area under the receiver operator characteristic curve of 0.71.^29^ Nonetheless, on adjusted analyses, the authors found that intermittent hypoxemic event characteristics did not reliably discriminate infants born extremely preterm with and without BPD-PH,^29^ suggesting a limited utility for these measures as independent predictors for BPD-PH in heterogeneous cohorts of infants born extremely preterm. Notably, in our study, the estimated change in probability of BPD-PH for a one-point increase in the RSS retained significance even after adjustment for gestational age and PMA at the time of echocardiogram (Table 2).

Given that exposure to mechanical ventilation and supplemental oxygen have both been implicated in the pathogenesis of BPD-PH, we speculate that the observed association between elevations in the RSS and an increased risk of BPD-PH has plausible criterion validity. Though specific mechanisms remain unknown, exposure of the developing preterm lung to a combination of ventilator-induced lung injury and oxidative stress likely contributes to impaired angiogenic signaling, endothelial progenitor cell dysfunction, and disrupted vascular growth in infants born extremely preterm who develop BPD-PH.^30–32^ Observational studies have identified chronic exposure to mechanical ventilation^4^ and supplemental oxygen^33^ as independent risk factors for the development of BPD-PH. Additionally, for those infants with established BPD-PH, the likelihood of discontinuation of pulmonary vasodilator therapy following NICU discharge is associated with a fewer number of days of invasive respiratory support, suggesting an earlier resolution of disease in those patients with earlier discontinuation of respiratory support.^34^ The results of these observational studies support clinical translational findings that demonstrate an imbalance of anti-angiogenic and pro-angiogenic factors in the developing lungs of infants born preterm who are exposed to mechanical ventilation and supplemental oxygen.^35–37^ While the associations observed in our study do not support a causal relationship between chronic exposure to mechanical ventilation/supplemental oxygen and BPD-PH, our results suggest that the RSS, which quantifies these exposures, may plausibly serve as a clinical risk score that can be measured longitudinally and used to monitor the risk of BPD-PH across the stages and spectrum of clinical disease.^7, 34, 38^ A particular strength of the RSS is that it can be measured across all levels of respiratory support that are used in the treatment of infants with BPD. Consequently, the score can plausibly be used to stratify BPD-PH risk over time as patients gradually wean from respiratory support or worsen in the setting of clinical deterioration.

Though there has been longstanding interest in identifying biochemical biomarkers that are associated with increased risk of BPD-PH, the diagnostic utility of BPD-PH biomarkers remains uncertain.^39^ Serum levels of BNP and NT-pro-BNP have been used to risk stratify adults and children with pulmonary arterial hypertension^40, 41^ as these peptides are released from the myocardium following wall stress and elevated levels of BNP and/or NT-pro-BNP may plausibly indicate right ventricular strain in the setting of pressure or volume overload.^42^ A limited number of retrospective, single-center studies have evaluated BNP and/or NT-pro-BNP as potential biomarkers for BPD-PH in infants born preterm with BPD.^43–48^ While these studies have observed significant elevations in BNP and/or NT-pro-BNP in infants with BPD-PH as compared to infants without BPD-PH,^44–46, 48^ interpretation of the diagnostic accuracy of these studies is limited due to their single-center retrospective designs, relatively small number of study subjects, and limited evaluation of BNP and/or NT-pro-BNP after 36 weeks’ PMA at timepoints concurrent with echocardiography.^39, 44–46, 49^ In the largest study evaluating the diagnostic accuracy of BNP for BPD-PH in infants with established BPD (i.e., after 36 weeks’ PMA) to date, Avitabile and colleagues identified a robust specificity but poor sensitivity for BNP relative to concurrent echocardiography suggesting a modest utility of BNP for the diagnosis and ongoing evaluation of BPD-PH.^48^

Published clinical practice guidelines recommend universal echocardiography screening for infants with BPD treated with positive airway pressure at 36 weeks’ PMA.^18, 50, 51^ Despite these recommendations, Lagatta and colleagues observed significant inter-center variation in echocardiogram utilization for the purpose of BPD-PH diagnosis among infants enrolled in the Children’s Hospital Neonatal Database with echocardiogram use rates ranging between 26–86% across the 23 centers that participated in this study.^4^ Echocardiograms are among the most utilized and expensive clinician-driven tests performed in the NICU^13^ and may be a potential target for expenditure reduction in healthcare spending associated with care provided to infants born extremely preterm.^52, 53^ To date, the cost-effectiveness of interval screening echocardiograms for infants with BPD-PH has not been studied. Though further studies are needed, we speculate that incorporation of a clinical BPD-PH risk score, such as the RSS, into BPD-PH screening recommendations may safely reduce the utilization of routine screening echocardiograms in low-risk infants (i.e., those with no prior history of BPD-PH and low RSS measurements) and precisely inform targeted BPD-PH screening in those infants at the highest risk of disease.

Our study has important limitations that should be considered. As this was a single-center study performed at a referral center with a dedicated BPD unit, it is possible that our results may not be generalizable. Given the retrospective observational design of the study, there is also the potential for incomplete confounding. Though we included model covariates known to be associated with both respiratory disease severity and BPD-PH, we acknowledge that unmeasured confounders could plausibly modify model effect estimates. Additionally, restriction of the cohort to infants born < 28 weeks’ gestation may contribute to selection bias in this study as we potentially excluded a relatively small proportion of infants born between 28–31 weeks’ gestation with BPD who develop BPD-PH after 36 weeks’ PMA.^4^ Lastly, as we defined BPD-PH dichotomously, we could not evaluate the association of RSS with BPD-PH severity. Our institution does not pursue cardiac catheterization to diagnose BPD-PH and so hemodynamic diagnostics that could be used to stratify BPD-PH severity were not available in this cohort. Given that the accuracy of echocardiographic assessments of BPD-PH severity is limited,^12, 18^ further studies, potentially in conjunction with advanced diagnostics including cardiac catheterization^12^ and cardiac magnetic resonance imaging,^54^ are needed to more precisely determine the association of RSS with BPD-PH severity.

In conclusion, our study demonstrated that elevations in the RSS were associated with an increased risk of BPD-PH. Measurement of the RSS approximate to the time of echocardiography may improve risk stratification for infants born extremely preterm who are evaluated for BPD-PH. Prospective studies that validate the RSS as a clinical susceptibility/risk biomarker are needed to identify the optimal timing of echocardiographic assessments for the evaluation and management of BPD-PH in infants born extremely preterm.

## Figures and Tables

**Table 1:** Clinical characteristics and outcomes of the cohort (n = 223) stratified by echocardiographic evidence of bronchopulmonary dysplasia-associated pulmonary hypertension (BPD-PH).

Characteristics			Overall	No BPD-PH	BPD-PH
N			223	155	68
Maternal race, n (%)	White		146 (65)	104 (67)	42 (62)
	Black		67 (30)	41 (26)	26 (38)
	Other		10 (5)	10 (7)	0 (0)
Maternal Hispanic ethnicity, n (%)			5 (2)	5 (3)	0 (0)
Maternal hypertensive disorder of pregnancy, n (%)			50 (22)	30 (19)	20 (29)
Maternal chorioamnionitis, n (%)			41 (18)	30 (24)	11 (16)
Receipt of antenatal corticosteroids, n (%)			190 (85)	130 (84)	60 (88)
Gestational age (weeks)			25 (24–26)	25 (24–26)	25 (24–26)
Birth weight (grams)			700 (592–865)	755 (615–890)	614 (515–775)
SGA, n (%)			33 (15)	10 (7%)	23 (34%)
Male sex, n (%)			132 (59)	95 (61%)	37 (54%)
Singleton birth, n (%)			166 (74)	118 (76)	74 (71)
1 Minute APGAR score, median (IQR)			3 (1–5)	3 (2–5)	3 (1–4)
5 Minute APGAR score, median (IQR)			6 (4–7)	6 (4–7)	5 (4–7)
Treatment with surfactant, n (%)			186 (83)	130 (84)	56 (82)
Grade III or IV interventricular hemorrhage, n (%)			26 (12%)	14 (9)	12 (18)
Periventricular leukomalacia, n (%)			17 (8%)	11 (7)	6 (9)
Retinopathy of prematurity, stage 2 or worse, n (%)			124 (56%)	82 (53)	42 (62)
Surgical necrotizing enterocolitis, n (%)			7 (3)	5 (3)	2 (3)
Pulmonary hypertension prior to 35 weeks’ PMA, n (%)			44 (20)	9 (6)	35 (51)
Treatment with inhaled nitric oxide prior to 36 weeks’ PMA, n (%)			19 (9)	12 (8)	7 (10)
Treatment with systemic steroids prior to 36 weeks’ PMA, n (%)			36 (16)	23 (15)	13 (19)
Respiratory support at 36 weeks’ PMA, n (%)		Nasal cannula ≤ 2 liters per minute	9 (4)	9 (6)	0 (0)
		Non-invasive positive airway pressure^a^	134 (56)	92 (59)	33 (49)
		Invasive mechanical ventilation	89 (40)	54 (35)	35 (51)
Length of stay (days)			177 (140–222)	164 (140–222)	204 (161–273)
Tracheostomy, n (%)			6 (3)	5 (3)	1 (2)
Deceased at discharge			8 (4)	1 (0.6%)	7 (10.3%)

**Abbreviations –** SGA – small for gestational age; PMA – post-menstrual age; BPD – bronchopulmonary dysplasia.

a Includes high-flow nasal cannula > 2 liters per minute flow, nasal continuous positive airway pressure, and non-invasive positive pressure ventilation.

**Table 2:** Percent change and odds ratio of bronchopulmonary-dysplasia associated pulmonary hypertension for one-point elevation in the respiratory severity score (RSS).

Outcome	Model 1^a^	Model 2^b^	Model 3^c^
Change in probability of BPD-PH^d^ (95% CI)	0.032 (0.021, 0.042)	0.032 (0.021, 0.042)	0.027 (0.017, 0.038)
Odds ratio (95% CI)	1.3 (1.2, 1.4)	1.3 (1.2, 1.4)	1.3 (1.2, 1.4)

**Abbreviations –** BPD-PH: bronchopulmonary dysplasia associated pulmonary hypertension

a Unadjusted model.

b Adjusted for gestational age.

c Adjusted for gestational age and post-menstrual age at the time of the echocardiogram.

d Change in probability using the average marginal effect (i.e. the difference in the probability of the outcome given the value of the covariate(s) in the corresponding model).

## Abbreviations

BPD: bronchopulmonary dysplasia
BPD-PH: bronchopulmonary dysplasia-associated pulmonary hypertension
PMA: post-menstrual age
NICU: neonatal intensive care unit
RSS: modified respiratory severity score
MAP: mean airway pressure
FiO_2_: fraction of inspired oxygen
NCH: Nationwide Children’s Hospital
PEEP: positive-end expiratory pressure
PIP: peak inspiratory pressure
t_i_: inspiratory time
t_e_: expiratory time
CPAP: continuous positive airway pressure
LPM: liters per minute
RVSP: right ventricular systolic pressure
BNP: brain natriuretic peptide
NT-pro-BNP: N-terminal pro b-type natriuretic peptide
IQR: interquartile range

## References

[1] Stoll BJ, Hansen NI, Bell EF, Walsh MC, Carlo WA, Shankaran S, Trends in Care Practices, Morbidity, and Mortality of Extremely Preterm Neonates, 1993–2012. JAMA. 2015;314:1039–51. 26348753 10.1001/jama.2015.10244 PMC4787615

[2] Khemani E, McElhinney DB, Rhein L, Andrade O, Lacro RV, Thomas KC, Pulmonary artery hypertension in formerly premature infants with bronchopulmonary dysplasia: clinical features and outcomes in the surfactant era. Pediatrics. 2007;120:1260–9. 18055675 10.1542/peds.2007-0971

[3] Slaughter JL, Pakrashi T, Jones DE, South AP, Shah TA. Echocardiographic detection of pulmonary hypertension in extremely low birth weight infants with bronchopulmonary dysplasia requiring prolonged positive pressure ventilation. J Perinatol. 2011;31:635–40. 21311503 10.1038/jp.2010.213

[4] Lagatta JM, Hysinger EB, Zaniletti I, Wymore EM, Vyas-Read S, Yallapragada S, The Impact of Pulmonary Hypertension in Preterm Infants with Severe Bronchopulmonary Dysplasia through 1 Year. J Pediatr. 2018;203:218–24 e3. 30172426 10.1016/j.jpeds.2018.07.035 PMC6460906

[5] Wu KY, Jensen EA, White AM, Wang Y, Biko DM, Nilan K, Characterization of Disease Phenotype in Very Preterm Infants with Severe Bronchopulmonary Dysplasia. Am J Respir Crit Care Med. 2020;201:1398–406. 31995403 10.1164/rccm.201907-1342OC PMC7258644

[6] Kim DH, Kim HS, Choi CW, Kim EK, Kim BI, Choi JH. Risk factors for pulmonary artery hypertension in preterm infants with moderate or severe bronchopulmonary dysplasia. Neonatology. 2012;101:40–6. 21791938 10.1159/000327891

[7] Altit G, Bhombal S, Hopper RK, Tacy TA, Feinstein J. Death or resolution: the “natural history” of pulmonary hypertension in bronchopulmonary dysplasia. J Perinatol. 2019;39:415–25. 30617286 10.1038/s41372-018-0303-8

[8] Mourani PM, Sontag MK, Younoszai A, Miller JI, Kinsella JP, Baker CD, Early pulmonary vascular disease in preterm infants at risk for bronchopulmonary dysplasia. Am J Respir Crit Care Med. 2015;191:87–95. 25389562 10.1164/rccm.201409-1594OC PMC4299632

[9] Arjaans S, Zwart EAH, Ploegstra MJ, Bos AF, Kooi EMW, Hillege HL, Identification of gaps in the current knowledge on pulmonary hypertension in extremely preterm infants: A systematic review and meta-analysis. Paediatr Perinat Epidemiol. 2018;32:258–67. 29341209 10.1111/ppe.12444

[10] Jobe AH, Bancalari E. Bronchopulmonary dysplasia. Am J Respir Crit Care Med. 2001;163:1723–9. 11401896 10.1164/ajrccm.163.7.2011060

[11] Jensen EA, Dysart K, Gantz MG, McDonald S, Bamat NA, Keszler M, The Diagnosis of Bronchopulmonary Dysplasia in Very Preterm Infants. An Evidence-based Approach. Am J Respir Crit Care Med. 2019;200:751–9. 30995069 10.1164/rccm.201812-2348OC PMC6775872

[12] Mourani PM, Sontag MK, Younoszai A, Ivy DD, Abman SH. Clinical utility of echocardiography for the diagnosis and management of pulmonary vascular disease in young children with chronic lung disease. Pediatrics. 2008;121:317–25. 18245423 10.1542/peds.2007-1583 PMC3121163

[13] King BC, Richardson T, Patel RM, Lee HC, Bamat NA, Patrick SW, Cost of clinician-driven tests and treatments in very low birth weight and/or very preterm infants. J Perinatol. 2021;41:295–304. 33268831 10.1038/s41372-020-00879-6

[14] Ballard RA, Truog WE, Cnaan A, Martin RJ, Ballard PL, Merrill JD, Inhaled nitric oxide in preterm infants undergoing mechanical ventilation. N Engl J Med. 2006;355:343–53. 16870913 10.1056/NEJMoa061088

[15] Kielt MJ, Logan JW, Backes CH, Conroy S, Reber KM, Shepherd EG, Noninvasive Respiratory Severity Indices Predict Adverse Outcomes in Bronchopulmonary Dysplasia. J Pediatr. 2022;242:129–36 e2. 34774575 10.1016/j.jpeds.2021.11.015

[16] Von Elm E, Altman DG, Egger M, Pocock SJ, Gøtzsche PC, Vandenbroucke JP, The Strengthening the Reporting of Observational Studies in Epidemiology (STROBE) Statement: guidelines for reporting observational studies. International journal of surgery. 2014;12:1495–9.25046131

[17] Kielt MJ, Logan JW, Backes CH, Conroy S, Reber KM, Shepherd EG, Non-Invasive Respiratory Severity Indices Predict Adverse Outcomes in Bronchopulmonary Dysplasia. J Pediatr. 2021. 10.1016/j.jpeds.2021.11.015 34774575

[18] Krishnan U, Feinstein JA, Adatia I, Austin ED, Mullen MP, Hopper RK, Evaluation and Management of Pulmonary Hypertension in Children with Bronchopulmonary Dysplasia. J Pediatr. 2017;188:24–34 e1. 28645441 10.1016/j.jpeds.2017.05.029

[19] Holmberg MJ, Andersen LW. Collider Bias. JAMA. 2022;327:1282–3. 35285854 10.1001/jama.2022.1820

[20] Goss KN, Beshish AG, Barton GP, Haraldsdottir K, Levin TS, Tetri LH, Early Pulmonary Vascular Disease in Young Adults Born Preterm. Am J Respir Crit Care Med. 2018;198:1549–58. 29944842 10.1164/rccm.201710-2016OC PMC6298636

[21] Abman SH, Hansmann G, Archer SL, Ivy DD, Adatia I, Chung WK, Pediatric Pulmonary Hypertension: Guidelines From the American Heart Association and American Thoracic Society. Circulation. 2015;132:2037–99. 26534956 10.1161/CIR.0000000000000329

[22] Backes CH, Cua C, Kreutzer J, Armsby L, El-Said H, Moore JW, Low weight as an independent risk factor for adverse events during cardiac catheterization of infants. Catheter Cardiovasc Interv. 2013;82:786–94. 23436647 10.1002/ccd.24726

[23] Sahni M, Yeboah B, Das P, Shah D, Ponnalagu D, Singh H, Novel biomarkers of bronchopulmonary dysplasia and bronchopulmonary dysplasia-associated pulmonary hypertension. J Perinatol. 2020;40:1634–43. 32811975 10.1038/s41372-020-00788-8 PMC7664991

[24] Mestan KK, Gotteiner N, Porta N, Grobman W, Su EJ, Ernst LM. Cord Blood Biomarkers of Placental Maternal Vascular Underperfusion Predict Bronchopulmonary Dysplasia-Associated Pulmonary Hypertension. J Pediatr. 2017;185:33–41. 28162769 10.1016/j.jpeds.2017.01.015 PMC5529237

[25] Abbas G, Shah S, Hanif M, Shah A, Rehman AU, Tahir S, The frequency of pulmonary hypertension in newborn with intrauterine growth restriction. Sci Rep. 2020;10:8064. 32415157 10.1038/s41598-020-65065-2 PMC7229189

[26] Check J, Gotteiner N, Liu X, Su E, Porta N, Steinhorn R, Fetal growth restriction and pulmonary hypertension in premature infants with bronchopulmonary dysplasia. J Perinatol. 2013;33:553–7. 23328924 10.1038/jp.2012.164 PMC3633609

[27] Bhat R, Salas AA, Foster C, Carlo WA, Ambalavanan N. Prospective analysis of pulmonary hypertension in extremely low birth weight infants. Pediatrics. 2012;129:e682–9. 22311993 10.1542/peds.2011-1827 PMC3289526

[28] Weismann CG, Asnes JD, Bazzy-Asaad A, Tolomeo C, Ehrenkranz RA, Bizzarro MJ. Pulmonary hypertension in preterm infants: results of a prospective screening program. J Perinatol. 2017;37:572–7. 28206997 10.1038/jp.2016.255

[29] Gentle SJ, Travers CP, Nakhmani A, Indic P, Carlo WA, Ambalavanan N. Intermittent Hypoxemia and Bronchopulmonary Dysplasia with Pulmonary Hypertension in Preterm Infants. Am J Respir Crit Care Med. 2023;207:899–907. 36449386 10.1164/rccm.202203-0580OC PMC10111996

[30] Baker CD, Abman SH, Mourani PM. Pulmonary Hypertension in Preterm Infants with Bronchopulmonary Dysplasia. Pediatr Allergy Immunol Pulmonol. 2014;27:8–16. 24669351 10.1089/ped.2013.0323 PMC3961769

[31] Hansmann G, Sallmon H, Roehr CC, Kourembanas S, Austin ED, Koestenberger M, Pulmonary hypertension in bronchopulmonary dysplasia. Pediatr Res. 2021;89:446–55. 32521539 10.1038/s41390-020-0993-4 PMC7979539

[32] Malloy KW, Austin ED. Pulmonary hypertension in the child with bronchopulmonary dysplasia. Pediatr Pulmonol. 2021;56:3546–56. 34324276 10.1002/ppul.25602 PMC8530892

[33] Aswani R, Hayman L, Nichols G, Luciano AA, Amankwah EK, Leshko JL, Oxygen requirement as a screening tool for the detection of late pulmonary hypertension in extremely low birth weight infants. Cardiol Young. 2016;26:521–7. 26119883 10.1017/S1047951115000608

[34] Avitabile CM, Zhang X, Ampah SB, Wang Y, Ash D, Nilan K, Factors associated with discontinuation of pulmonary vasodilator therapy in children with bronchopulmonary dysplasia-associated pulmonary hypertension. J Perinatol. 2022;42:1246–54. 35676536 10.1038/s41372-022-01421-6

[35] Baker CD, Balasubramaniam V, Mourani PM, Sontag MK, Black CP, Ryan SL, Cord blood angiogenic progenitor cells are decreased in bronchopulmonary dysplasia. Eur Respir J. 2012;40:1516–22. 22496315 10.1183/09031936.00017312 PMC5596882

[36] De Paepe ME, Greco D, Mao Q. Angiogenesis-related gene expression profiling in ventilated preterm human lungs. Exp Lung Res. 2010;36:399–410. 20718599 10.3109/01902141003714031

[37] De Paepe ME, Patel C, Tsai A, Gundavarapu S, Mao Q. Endoglin (CD105) up-regulation in pulmonary microvasculature of ventilated preterm infants. Am J Respir Crit Care Med. 2008;178:180–7. 18420967 10.1164/rccm.200608-1240OC PMC2453512

[38] Aoyama BC, McGrath-Morrow SA, Collaco JM. Characteristics of Children with Bronchopulmonary Dysplasia with Prolonged and/or Later-Onset Pulmonary Hypertension. Ann Am Thorac Soc. 2021;18:1746–8. 33770446 10.1513/AnnalsATS.202012-1471RL PMC8522294

[39] Xiong T, Kulkarni M, Gokulakrishnan G, Shivanna B, Pammi M. Natriuretic peptides in bronchopulmonary dysplasia: a systematic review. J Perinatol. 2020;40:607–15. 31925319 10.1038/s41372-019-0588-2

[40] Yildiz M, Sahin A, Behnes M, Akin I. An Expanding Role of Biomarkers in Pulmonary Arterial Hypertension. Curr Pharm Biotechnol. 2017;18:491–4. 28641568 10.2174/1389201018666170615082510

[41] Fijalkowska A, Kurzyna M, Torbicki A, Szewczyk G, Florczyk M, Pruszczyk P, Serum N-terminal brain natriuretic peptide as a prognostic parameter in patients with pulmonary hypertension. Chest. 2006;129:1313–21. 16685024 10.1378/chest.129.5.1313

[42] Lewis RA, Durrington C, Condliffe R, Kiely DG. BNP/NT-proBNP in pulmonary arterial hypertension: time for point-of-care testing? Eur Respir Rev. 2020;29. 10.1183/16000617.0009-2020 PMC9488846 32414745

[43] Dasgupta S, Aly AM, Malloy MH, Okorodudu AO, Jain SK. NTproBNP as a surrogate biomarker for early screening of pulmonary hypertension in preterm infants with bronchopulmonary dysplasia. Journal of Perinatology. 2018;38:1252–7. 29977013 10.1038/s41372-018-0164-1

[44] Montgomery AM, Bazzy-Asaad A, Asnes JD, Bizzarro MJ, Ehrenkranz RA, Weismann CG. Biochemical screening for pulmonary hypertension in preterm infants with bronchopulmonary dysplasia. Neonatology. 2016;109:190–4. 26780635 10.1159/000442043

[45] Cuna A, Kandasamy J, Sims B. B-type natriuretic peptide and mortality in extremely low birth weight infants with pulmonary hypertension: a retrospective cohort analysis. BMC pediatrics. 2014;14:1–6. 24612708 10.1186/1471-2431-14-68 PMC3975241

[46] Cuna A, Kandasamy J, Fineberg N, Sims B. B-type natriuretic peptide is a biomarker for pulmonary hypertension in preterm infants with bronchopulmonary dysplasia. Res Rep Neonatology. 2013;3:33–6.

[47] Amdani SM, Mian MUM, Thomas RL, Ross RD. NT-pro BNP—A marker for worsening respiratory status and mortality in infants and young children with pulmonary hypertension. Congenital heart disease. 2018;13:499–505. 29575641 10.1111/chd.12601

[48] Avitabile CM, Ansems S, Wang Y, Fraga MV, Kirpalani HM, Zhang H, Accuracy of Brain Natriuretic Peptide for Diagnosing Pulmonary Hypertension in Severe Bronchopulmonary Dysplasia. Neonatology. 2019:1–7. 10.1159/000499082 31096210

[49] Dasgupta S, Aly AM, Malloy MH, Okorodudu AO, Jain SK. NTproBNP as a surrogate biomarker for early screening of pulmonary hypertension in preterm infants with bronchopulmonary dysplasia. J Perinatol. 2018;38:1252–7. 29977013 10.1038/s41372-018-0164-1

[50] Abman SH, Ivy DD, Archer SL, Wilson K, Committee AAJGfPPH. Executive Summary of the American Heart Association and American Thoracic Society Joint Guidelines for Pediatric Pulmonary Hypertension. Am J Respir Crit Care Med. 2016;194:898–906. 27689707 10.1164/rccm.201606-1183ST PMC5074658

[51] Abman SH, Collaco JM, Shepherd EG, Keszler M, Cuevas-Guaman M, Welty SE, Interdisciplinary Care of Children with Severe Bronchopulmonary Dysplasia. J Pediatr. 2017;181:12–28 e1. 27908648 10.1016/j.jpeds.2016.10.082 PMC5562402

[52] Rolnitsky A, Urbach D, Unger S, Bell CM. Regional variation in cost of neonatal intensive care for extremely preterm infants. BMC Pediatr. 2021;21:134. 33731048 10.1186/s12887-021-02600-8 PMC7968295

[53] Beam AL, Fried I, Palmer N, Agniel D, Brat G, Fox K, Estimates of healthcare spending for preterm and low-birthweight infants in a commercially insured population: 2008–2016. J Perinatol. 2020;40:1091–9. 32103158 10.1038/s41372-020-0635-z PMC7314662

[54] Critser PJ, Higano NS, Tkach JA, Olson ES, Spielberg DR, Kingma PS, Cardiac Magnetic Resonance Imaging Evaluation of Neonatal Bronchopulmonary Dysplasia-associated Pulmonary Hypertension. Am J Respir Crit Care Med. 2020;201:73–82. 31539272 10.1164/rccm.201904-0826OC PMC6938152

